# The Effect of Natural Antioxidants on Quality and Shelf Life of Beef and Beef Products

**DOI:** 10.17113/ftb.57.04.19.6267

**Published:** 2019-12

**Authors:** Olaf K. Horbańczuk, Marcin A. Kurek, Atanas G. Atanasov, Mladen Brnčić, Suzana Rimac Brnčić

**Affiliations:** 1 Department of Technique and Food Development, Warsaw University of Life Sciences (WULS-SGGW), Nowoursynowska Street 159 c, 02-776 Warsaw, Poland; 2 Institute of Genetics and Animal Breeding Polish Academy of Sciences, Jastrzębiec Postępu 36A Street, 05-552 Magdalenka, Poland; 3 Department of Pharmacognosy, University of Vienna, Althanstrasse 14, 1090 Vienna, Austria; 4 Faculty of Food Technology and Biotechnology, University of Zagreb, Pierottijeva 6, 10000 Zagreb, Croatia

**Keywords:** natural antioxidants, beef products, meat quality, shelf life

## Abstract

Oxidation processes are the major cause of deterioration of meat quality and shelf life of meat products, leading to negative changes in nutritive value and in sensory and physicochemical properties of meat. Until now, the synthetic antioxidants like butylated hydroxyl anisole have been commonly used to prevent oxidation, which however may cause potential human health risks and increase toxicity of the product. However, natural antioxidants can be the alternative solution for this problem since plants and plant materials are rich in bioactive compounds (as natural antioxidants) with potential health beneficial effects. Moreover, the interest of consumers in using natural products is still increasing. There is an expectation of replacing synthetic antioxidants and preservatives by natural ones. Therefore, the aim of the present review is to provide information on the effect of natural antioxidants from vegetables and fruits like olives, pomegranate or grapes, and herbs and spices like rosemary, oregano, sage, black cumin or turmeric, rich in bioactive compounds on quality and shelf life of beef and beef products.

## INTRODUCTION

Lipid and protein oxidation are the major cause of deterioration of meat quality and shelf life limitations of meat products. It leads to unfavourable changes in nutritive value and in sensory and physicochemical properties of meat like colour, flavour or tenderness (*1*-*6*). The oxidative deterioration of meat can be reduced with the addition of synthetic or natural antioxidants. However, the use of synthetic antioxidants like butylated hydroxytoluene (BHT) or butylated hydroxyanisole (BHA) has been restricted because of possible health risks and toxicity (*7*). Thus, there is increased consumer interest in using natural products such as antioxidants, vitamins, minerals or fibre from plants and plant materials which are rich in bioactive compounds (*e.g.* phenolic compounds) to preserve and improve meat. Hydrocolloids could also be used in meat products to improve functional properties of meat in the way of salt reduction and stability during freezing (*8*, *9*). Aside from antioxidants, plants also contain a range of other bioactive compounds with potential beneficial health effects (*10*-*15*). Plants rich in antioxidants include vegetables and fruits like olives, pomegranate, grapes, berries (*16*-*21*), as well as herbs and spices like rosemary, oregano, broccoli, sage, black cumin, thyme or turmeric (*22*-*24*). Most of the plants have a relatively high nutrient (biological peptides, polyunsaturated fatty acids, calcium, iron or phosphorus) content and antimicrobial properties, which is overall very important for their health benefits (*25*, *26*). It should be emphasised that the concentration of antioxidant compounds in plant sources varies considerably depending on the plant and also their amount and application form in diets and meat products is different (*27*, *28*). High amounts may lead to unfavourable effects through pro-oxidative processes (*29*, *30*). Technological strategies involve the application of antioxidants directly into the meat and meat products or coating of packaging materials with plants in different form to improve the oxidative stability of the products. In this paper, we focus on the effect of selected plants and plant materials as natural antioxidants rich in bioactive molecules on the quality and shelf life of beef and beef products.

## NATURAL ANTIOXIDANT ADDITIVES

### Additives obtained from fruits and vegetables

Pomegranate aril and mesocarp contain a high concentration of antioxidants compared to blueberry, grapes and green tea (*31*). The peel and seeds are rich sources of tannins and anthocyanins (*31*, *32*). Hydrolysable tannins cover mainly bioactive molecules like ellagitannins, gallotannins and punicalgin, whereas anthocyanins include delphinindin, cyanidin and pelargonidin (*32*). El-Nashi *et al.* (*33*) assessed the impact of pomegranate peel powder addition (1, 2 or 3%) on quality of beef sausage during storage at 4 °C for 12 days. The addition of various mass fractions of pomegranate peel powder increased storage stability and reduced values of *tert*-butyl alcohol (TBA) of beef sausage during refrigerated storage and improved the microbiological safety. This could be the effect of phenolic compounds present in pomegranate peel. The effect of pomegranate peel extract on the delay of lipid and protein oxidation in beef meatballs was also investigated during frozen storage at -18 °C by Turgut *et al*. (*34*). It is interesting that the addition of peel extract of up to 1.0% to the meatballs limited the oxidation more than a synthetic antioxidant like BHT, probably due to the high content of polyphenols in the peel extract (*34*, *35*). Also, the addition of 1.0% peel extract maintained the colour intensity. The results of sensory analyses revealed that its addition to meatballs was effective in preventing rancid odour formation. The effect of amount, storage temperature and time of exposure of the various natural antioxidants on lipid and protein oxidation in beef and beef products is shown in the Table 1 (*35*-*48*). Garcia-Lomillo *et al*. (*36*) studied the effect of pomace, rich in phenolics and stilbenes like resveratrol (*49*), on protein oxidation in beef patties during high oxygen atmosphere storage. For gaining a wider perspective, the ability of red and white grape pomace with 9.9 and 4.0 mg phenols respectively was tested. The results showed that red grape pomace protected against protein radical formation, as opposed to white grape pomace, thus it could be added as an alternative to artificial antioxidants, *e.g*. the sulfite used in meat products. A later study (*50*) assessed the ability of pomace (used as three different types of seasoning) to inhibit lipid oxidation in raw and cooked beef patties. All types of seasonings showed different effectiveness in inhibiting lipid oxidation, thus the seedless red grape pomace was the most effective, significantly inhibiting the formation of volatile organic compounds (*e.g.* hexanal, 1-pentanol or 1-hexanol). That study also showed the potential capacity of grape pomace to delay the formation of rancid odour during storage, suggesting that it could be used as a natural alternative to extend the shelf-life of meat products.

**Table 1:** Effect of dose/concentration, storage temperature and time of exposure of the natural antioxidants on lipid and protein oxidation in beef and beef meat products

Natural antioxidant	Amount added	Type of meat	Storage temperature/°C	*t*(storage)/day	Effect	Reference
Pomegranate peel extract	1%	beef meatballs	4±1	0, 2, 4, 6, 8	Effective prevention of rancid odour formation, noticeable antioxidant activity	(*35*)
Wine pomace	2 g/100 g	refrigerated beef patty	4	0, 4, 8, 12, 15	Inhibition of lipid oxidation, capacity to delay the formation of rancid odour during storage	(*36*)
Plum juice concentrate	2.5 and 5%	roast beef	<4	0, 2, 4, 6, 8, 10	Lower level of TBARS, reduction of lipid oxidation	(*37*)
Grape seed extract	50, 200 and 1000 mg/kg	minced beef	4±1	0, 2, 4, 8, 10	Significantly lower values of pH, TBARS and TBC than those of control and BHT-treated samples, enhancement of shelf life	(*38*)
Green tea extract	300 mg/100 g	raw beef patty	4	0, 3, 6, 9	Inhibition of lipid oxidation, lower microbial spoilage, delayed redness loss	(*39*)
Olive leaf extract and powder	100 and 150 µg/g	minced beef	4	0, 3, 6, 9	Decrease of storage loss and defrosting loss, inhibition of lipid oxidation and myoglobin oxidation	(*40*)
Olive leaf extract	100 and 200 µg/g	minced beef patty	4	9, 12	Reduction of TBARS value in raw beef patty in packaging systems, reduction of oxymyoglobin oxidation	(*41*)
Garlic extract	50 mg/100 g	ground beef	4	1, 3, 5, 7, 9, 11, 13	Protection of phospholipids, fatty acids and polypeptides from oxidation, stabilization of the redness in raw meat	(*42*)
Mulberry extract	0, 100, 500 and 1000 µg/g	raw ground beef	4	0, 6, 12 h and 1, 2, 3, 4, 7, 10, 13	Extension of shelf-life, maintaining colour, reduction of peroxide and thiobarbituric acid reactive substance values during storage	(*43*)
Oregano and sage essential oil	3%	raw beef	4	12	High antioxidant effect/ reduction of oxidation	(*44*)
Pine bark, grape seed, rosemary extract	1%	cooked beef	4	3, 9	Highly antioxidant and antimicrobial activity	(*45*)
Cinnamon and clove	250 mg/100 g	ground beef	5	2, 4, 6	Cloves showed higher antioxidant potential than BHA, positive effect on decreasing TBARS	(*46*)
Ginger rhizome extract and fenugreek seeds	0.5 mg/1 g	ground beef patty	5, 25, 37	0, 7, 14, 21	Effective in retarding rancid odour, TBA and colour change, control of lipid oxidation	(*47*)
*Moringa oleifera* and *Bidens pilosa* leaf extract	0.5 g and 1 g/kg	raw ground beef	4	0, 3, 6	Protection against lipid oxidation during storage, decrease of pH	(*48*)

Nuñez de Gonzalez *et al*. (*37*) reported a decreased lipid oxidation in precooked roast beef when treated with fresh and dried plum juice concentrates. The authors showed that the 5% addition of the fresh plum juice concentrate induced the lowest level of TBARS (thiobarbituric acid reactive substances) expressed as malonyldialdehyde (MDA) to 0.16 mg/kg as compared to the control group (0.62 mg/kg). There was no significant impact of plum juice addition on the colour or flavour of meat, which is important since the observable differences in the colour of meat may lead consumers to believe that the product is not properly processed and safe to consume. Gómez *et al*. (*51*) studied the shelf life and oxidative stability of refrigerated raw ground beef enriched with omega-3 fatty acids with grape seed extract used as inhibitor of lipid oxidation. Grape seed extract addition prevented rancidity in enriched ground beef, decreasing the oxidation value without affecting meat colour and odour. The results suggest that grape seed extract can be a technologically viable alternative to synthetic stabilisers for preventing the lipid oxidation in new fresh or functional meat products.

The impact of grape seed extract on sensory characteristics, inhibition of lipid oxidation and bacterial growth in raw minced beef during refrigerated storage at 4 °C for 10 days was studied by Amin and Edris (*38*). The authors compared the addition of different amounts of grape seed extract with the synthetic antioxidant BHT. The results showed that grape seed extract is efficient with a concentration-dependent activity. Minced beef samples treated with grape seed extract showed significantly lower values of pH, TBARS and total bacterial counts than the control samples and samples treated with BHT during refrigerated storage. Grape seed extract confers proper protection against lipid oxidation and microbial spoilage, thus, it could be used as an alternative to synthetic antioxidants, *e.g*. BHT. In this line, the authors concluded that grape seed extract can be used as both natural antioxidant and antibacterial agent during refrigerated storage of meat. Also, Bañón *et al.* (*39*) evaluated the antioxidant and antimicrobial activities of grape seed extract and green tea in raw beef patty. Their work showed that either green tea or grape seed extract had preservative effects on beef patty, especially protecting against meat oxidation, and also had positive effect on sensory properties.

DeJong and Lanari (*52*) assessed the antioxidant activity of extracts from olive oil in beef and compared the results with those of green tea and red wine. They showed that polyphenolic extracts from the olive oil reduced the TBARS values in precooked beef (63-83%), but compared to other natural antioxidants, tea has the highest antioxidant potential, followed by olive oil, and red wine with the lowest antioxidant potential. It should be noted that the main component of virgin olive oil and olive waste is hydroxytyrosol, which is a type of phenolic phytochemical with strong antioxidant properties (*53*). Furthermore, important nutrients in olive oil are free fatty acids, aliphatic alcohols, tocopherols, hydrocarbons, sterols, triterpenic compounds and pigments (*54*). Therefore, the olive oil extract has an excellent potential and significantly inhibits lipid oxidation in ground beef (*52*).

Aouidi *et al*. (*40*) investigated the antioxidant potential of olive leaves added to minced beef. The results showed that olive leaves have an ability to inhibit (p<0.05) lipid oxidation (TBARS values were reduced by 25-65%) and myoglobin oxidation. They also observed that olive leaves decreased the mass loss of minced beef after defrosting and during storage. Addition of olive leaves had no impact on sensory properties of meat. The authors concluded that the addition of olive leaves could enhance the stability of the beef products and extend storage time.

Hayes *et al*. (*41*) investigated the effect of olive leaf extract on the quality and shelf-life stability of packaged raw minced beef patty. The olive leaf extract reduced TBARS values and significantly reduced oxymyoglobin oxidation in the packaged raw patty. Moreover, olive leaf extract increased antibacterial and antifungal activities against Gram-positive bacteria (*e.g. Helicobacter pylori* or *Staphylococcus aureus*), Gram-negative bacteria (*e.g. Campylobacter jejuni*) and fungi, as shown by Sudjana *et al*. (*55*).

Zhang *et al*. (*42*) studied the effect of garlic extract on colour, lipid oxidation and oxidative breakdown products in raw ground beef during refrigerated storage. Allicin is a thiosulfinate extract of garlic, recognized as a very strong antioxidant, that exhibits a range of other interesting bioactivities (*56*). Also, garlic is rich in selenium, allyl cysteine and allyl disulfide. It was demonstrated that garlic extracts protected phospholipids, fatty acids and polypeptides from oxidation. Moreover, garlic extracts could stabilize the redness in raw ground beef during refrigerated storage due to antioxidant properties which prevent the oxidation of oxymyoglobin. In following investigations, Zhang *et al*. (*43*) investigated the impact of mulberry leaf extract on the colour, lipid oxidation and antioxidant activity of enzymes in raw ground beef during storage. Mulberry leaves are rich in quercetin 3-(6”-malonylglucoside), rutin and isoquercetin. They observed that mulberry leaf extract can be used as natural antioxidant to maintain the meat quality, *e.g*. colour, extend shelf-life and prevent economic loss for food processing industry. Moreover, the results showed that it is better to combine the mulberry leaf extract with vitamin E to maintain better quality of meat during storage.

### Additives obtained from herbs and spices

Fasseas *et al*. (*44*) determined the antioxidant activity of ground beef and pork meat homogenized with oregano and sage (3% essential oil) during 12 days of storage. Sage and rosemary contain phenolic compounds and are rich in carnosic acid, carnosol and rosmarinic acid. Sage contains high amounts of compounds with diverse bioactivities, such as flavonoids, diterpenoids triterpenes and steroids including rosmarinic acid or picein, 6-O-(E)-feruloyl-(α and β)-glucopyranoside (*57*, *58*). Before storage, both types of raw ground meat samples were kept at 4 °C for 30 min and then cooked at 85 °C for 30 min. The results demonstrated that the sage and oregano significantly reduced the oxidation (*44*). The reported data demonstrated that oregano is more effective as antioxidant in both types of meat than sage, as demonstrated by TBA assay. Moreover, much higher TBA values were measured in the cooked meat than in the raw samples. According to the authors, higher antioxidant effect was observed in cooked than in raw meat and the meat proteins greatly affected the antioxidant activity. Oregano may be used as a natural antioxidant in meat products, but current research shows that grape seed, cranberry and sage extracts, among others, which exhibit a more significant reduction in TBARS values in a variety of products, are more powerful natural antioxidants (*44*).

Rosemary contains very high amounts of antioxidants, including phenolic compounds (*e.g.* carnosic acid (*59*), carnosol and rosmarinic acid, caffeic acid and flavonoids), which have been associated with the high antioxidant activity (*60*-*63*). Lund *et al*. (*64*) investigated the influence of rosemary extract in combination with modified atmosphere packaging on protein and lipid oxidation in beef patty during storage up to 6 days at 4 °C. They demonstrated that in high oxygen atmosphere this antioxidant protected the natural red colour in beef.

In turn, Fernandez-Lopez *et al*. (*65*) studied the antioxidant and antibacterial effects of rosemary, lemon and orange extracts in beef meatballs. The authors showed the positive effect of the addition of rosemary and citrus extract to beef meatballs without negative impact on the acceptability of the product. Sensory analysis, especially aroma and acceptability, indicated the significant advantages of application of rosemary and citrus extracts in beef products (*66*). Green tea extract was studied as a source of antioxidant and antimicrobial compounds that have the potential to improve the overall quality and extend the shelf life of beef. Tea catechins at 200-400 mg/kg can be used as inhibitor of lipid oxidation with a significant effect on beef, reaching much greater effect than vitamin C (*67*). Moreover, treatments with tea catechins and vitamin C had a significant effect on lipid oxidation in cooked beef (0.32 mg MDA per kg of meat) compared to beef control sample without these supplements (>1.1 mg MDA per kg meat). According to the authors, green tea extract could be a proper antioxidant solution for the meat producers, comparable to synthetic alternatives.

The influence of rosemary oil, pine bark extract, grape seed extract and butylated hydroxyanisole/butylated hydroxytoluene (BHA/BHT) on microbial growth, colour change and lipid oxidation was analyzed in ground beef by Ahn *et al*. (*45*). The study demonstrated that 1.0% grape seed or pine bark extract rapidly reduced the numbers of *E. coli* in the first three days of storage, whereas 1.0% pine bark extract most effectively inhibited the increase of *E. coli* or *L. monocytogenes* after nine days of storage, followed by the grape seed extract and rosemary extract. Among the natural extracts used in that study, rosemary oil showed lower inhibitory effects against the pathogens than did grape seed extract and pine bark extract. Moreover, the retention of the red colour of cooked beef treated with pine bark and grape seed extracts was observed, and it was concluded that it may originate from the antioxidant effects and contribution of these extracts to pigments (*45*). It should be stressed that pine bark extract is a suitable source of phenolic compounds like procyanidins or condensed tannins, which have been shown to possess antioxidant activity (*68*, *69*).

The effect of rosemary and oregano extracts, added individually or in combination, and butylated hydroxyanisole/butylated hydroxytoluene (BHA/BHT), on lipid oxidation and fatty acid composition of irradiated beef burgers was investigated by Trindade *et al*. (*69*). The results of the experiment showed that rosemary extract, applied alone and in combination with either BHA/BHT or oregano extract, was more effective in maintaining a low oxidation level in the samples than the oregano extract used individually or in combination with BHA/BHT, and the antioxidant capacity of natural extracts decreased while lipid oxidation increased with storage time. Among the natural additives studied, the highest antioxidant capacity was obtained with rosemary extract.

In turn, Rojas and Brewer (*70*) evaluated the potential of oregano essence on the oxidation stability of cooked and refrigerated beef and demonstrated that the addition of 0.02% oregano essence was effective in decreasing the lipid oxidation in the meat. Oregano has antibiotic and antioxidant effects, along with other biological activities (*57*, *71*).

Jayathilakan *et al*. (*46*) compared the effect of natural antioxidants like cinnamon (cinnamaldehyde and eugenol in cinnamon oil were identified as the most active antibacterial components) and cloves with synthetic one like BHA in beef ground meat samples purchased in local store, packed in polypropylene bags, cooked in a water bath under atmospheric pressure for 35 min and then stored at 5 °C for 6 days. Clove is a source of eugenol, which is reported to have antifungal activity (*72*), and also β-caryophyllene, α-humulene (2.1%) and eugenyl acetate (*73*). The authors reported that either cinnamon or cloves had positive effect (p>0.05) on the decrease of lipid oxidation, manifested as lower TBARS value in cooked ground beef. No difference was observed (p>0.05) between the samples of ground beef treated with cinnamon and those treated with 0.02% BHA. It is interesting that cloves showed higher antioxidant potential (p>0.05) than BHA. The authors stated that cloves had antioxidant potential similar to the synthetic antioxidant BHA and could be applied as a natural substitute to increase shelf life of beef products.

Mizi *et al*. (*74*) investigated the combined effect of sage (0.3 and 0.6%) and high pressure processing on the antimicrobial and antioxidant quality of beef burgers during prolonged storage (up to 60 days). The results showed that lipid oxidation was higher in all samples during storage but sage powder can be recognized as an effective antioxidant, retarding lipid oxidation during 60 days of chilled storage in the beef burgers processed under high pressure. Moreover, the microbial quality of the burgers was evaluated as acceptable. The application of both treatments to obtain burgers with better oxidation and microbiological stability during prolonged storage without any effect on sensory attributes could be considered as an alternative way.

Mansour and Khalil (*47*) evaluated the antioxidant activity of ginger, potato peel and fenugreek seed extracts in ground beef patty. Ginger is rich in compounds like gingerols (*e.g*. 6-gingerdiol) and has antimicrobial and antioxidant potential, along with diverse additional bioactivities (*75*-*77*), whereas fenugreek seeds are rich in flavonoids, *e.g.* vitexin, tricin, naringenin, quercetin and tricin 7-O-β-d-glucopyranoside (*78*). Mansour and Khalil (*47*) showed that ginger rhizome extract exhibited the highest antioxidant activity and had an activity comparable to commercial antioxidants. Moreover, the extracts from ginger rhizome and fenugreek seeds added to beef patties were more effective than potato peel extract in controlling lipid oxidation and colour changes during storage (*47*).

Falowo *et al*. (*48*) examined the effect of leaf extracts of *Moringa oleifera* and *Bidens pilosa* on lipid oxidation and pH value of fresh beef during storage. The results showed that the application of both extracts can protect beef against oxidation during storage. The *B. pilosa* extract showed higher antioxidant activity (at 0.5 and 1 g/kg) than the *M. oleifera*, and it is mainly because *B. pilosa* leaves contained higher amounts of bioactive substances (*e.g*. tetradecanoic acid or dl-alpha-tocopherol) and had higher antioxidant activity (p<0.05). Furthermore, this study showed that both extracts are rich in phytochemicals with significant free radical scavenging activity.

Interesting research was also conducted by Van Hecke *et al*. (*78*), who assessed the antioxidant capacity of typical herbs like rosemary, and spices like turmeric and garlic during cooking of beef product and during *in vitro* digestion. The additions to the meat product were made separately either before or after heating, as a seasoning. The addition of herbs and spices before heating of the meat was more effective than after heating for reduction of oxidation during digestion. Herbs and turmeric showed higher antioxidant activity than other investigated additives (*73*, *78*).

This review demonstrates the potential of commonly utilized herbs and spices to reduce the lipid oxidation in meat and meat products. A reduction of the lipid oxidation product formation in meat is considered beneficial for human health (*79*-*83*), while the prevention of the formation of lipid oxidation products can reduce the risk of developing diseases of civilization related to a common diet rich in processed red meat (*84*).

## CONCLUSIONS

Natural antioxidants, including a variety of fruits, vegetables, herbs and spices added to meat and its products have multiple functions. They may play an antioxidant, antimicrobial and preservative role in beef and beef products during processing and storage, since plants and plant materials are rich in bioactive compounds. These compounds have the potential to inhibit or delay oxidation, which can be widely used by the food industry. Among culinary herbs and spices, the best protection capacity of the beef and beef products was found when applying sage and rosemary, especially for oxidation and colour stability. In turn, grape seeds, olive oil and leaves, characterized by the high amount of polyphenols, also showed similar positive antioxidant effect on beef and beef products during storage. In light of this, the mentioned antioxidants could be considered as a natural alternative to synthetic preservatives of the meat products.
